# Chitinase Gene Positively Regulates Hypersensitive and Defense Responses of Pepper to *Colletotrichum acutatum* Infection

**DOI:** 10.3390/ijms21186624

**Published:** 2020-09-10

**Authors:** Muhammad Ali, Quan-Hui Li, Tao Zou, Ai-Min Wei, Ganbat Gombojav, Gang Lu, Zhen-Hui Gong

**Affiliations:** 1 College of Horticulture, Northwest A&F University, Yangling 712100, China; alinhorti@yahoo.com; 2 Department of Horticulture, College of Agriculture and Biotechnology, Zhejiang University, Hangzhou 310058, China; smile_zoutao@163.com; 3 Qinghai Academy of Agricultural and Forestry Sciences, Xining, Qinghai 810016, China; liquanhui_2008@163.com; 4 Tianjin Vegetable Research Center, Tianjin 300384, China; waimin163@163.com; 5 School of Animal Sciences and Biotechnology, Mongolian University of Life Sciences, Ulaanbaatar 17024, Mongolia; ganbat30@yahoo.com

**Keywords:** *CaChiIII7*, cell-death, chitinase, *Colletotrichum acutatum*, pepper, plant defense, ROS burst, VIGS

## Abstract

Anthracnose caused by *Colletotrichum acutatum* is one of the most devastating fungal diseases of pepper (*Capsicum annuum* L.). The utilization of chitin-binding proteins or chitinase genes is the best option to control this disease. A chitin-binding domain (CBD) has been shown to be crucial for the innate immunity of plants and activates the hypersensitive response (HR). The *CaChiIII7* chitinase gene has been identified and isolated from pepper plants. *CaChiIII7* has repeated CBDs that encode a chitinase enzyme that is transcriptionally stimulated by *C. acutatum* infection. The knockdown of *CaChiIII7* in pepper plants confers increased hypersensitivity to *C. acutatum*, resulting in its proliferation in infected leaves and an attenuation of the defense response genes *CaPR1*, *CaPR5*, and *SAR8.2* in the *CaChiIII7*-silenced pepper plants. Additionally, H_2_O_2_ accumulation, conductivity, proline biosynthesis, and root activity were distinctly reduced in *CaChiIII7*-silenced plants. Subcellular localization analyses indicated that the *CaChiIII7* protein is located in the plasma membrane and cytoplasm of plant cells. The transient expression of *CaChiIII7* increases the basal resistance to *C. acutatum* by significantly expressing several defense response genes and the HR in pepper leaves, accompanied by an induction of H_2_O_2_ biosynthesis. These findings demonstrate that *CaChiIII7* plays a prominent role in plant defense in response to pathogen infection.

## 1. Introduction

Plants have innate defense systems to combat microbial pathogens. Terrestrial plants initially defend themselves from opportunistic aggressors by adopting structural ability, specialized sophisticated defensive response mechanisms, and genetically controlled regulatory pathways [1]. The plant immune system is often based on pattern-triggered immunity (PTI) and effector-triggered immunity (ETI) that provide an instant response and the subsequent activation of defense machinery to maintain survival [2]. However, the identification of invading pathogenic microorganisms and a complex transduction system acts to activate cellular protection in plants to block further pathogenic invasion and enhance plant resistance. In addition, plants recognize disease agents through transmembrane pattern recognition receptors (PRRs) or via intracellular proteins of the nucleotide-binding domain (NBD) leucine-rich repeat (NLR) superfamily that occurs inside the plant cell [3,4]. The introduction of R-type genes (resistance genes) via genetic engineering into plants can provide broad spectrum resistance and render them even more effective at avoiding infection and predation by microbes and pests, respectively [5]. To date, novel R genes are being identified and have confirmed diverse resistance specialties in various species [6]. This approach may exploit the mechanistic understanding and functional domain sites of resistance genes, which will help to generate genetic resistance and create possible opportunities to develop an innate immune response.

Triggering the receptors leads to defense reactions that are attentively orchestrated with sequence alterations at the cellular level. However, a series of signaling events are initiated to trigger early cellular responses after pathogens are recognized, such as variations in ion fluxes, salicylic acid (SA), abscisic acid (ABA), jasmonic acid (JA), nitric oxide (NO), and hydrogen peroxide (H_2_O_2_). These compounds are involved in the regulation of numerous defense genes, while saccharides (glucose and fructose) and sucrose are the key signals in the regulation of these signaling molecules during the protection of host plants from pathogenic fungi [7]. Previous studies suggested that the existence of sucrose permits the plants to develop efficient mechanisms of defense, including those against systemic fungal pathogens, such as *Fusarium oxysporum* [8,9,10]. Moreover, trehalose may also be a signal molecule that triggers the defense responses of plants against pathogenic fungi [11]. Furthermore, the accumulation of reactive oxygen species (ROS), such as H_2_O_2_, is associated with defense signaling and plant programmed cell death (PCD) [12,13]. Thus, the ROS, transcriptional alteration that includes chitinase genes, and the quick cell death response, known as the hypersensitive response (HR) [14,15,16]. These responses are also essential to hinder the growth/expansion of pathogens by encouraging cross-linking between the cell wall components and the mediation of signal transduction cascades that activate stress response and other types of defense genes [17]. Pepper genes that participate in cell death or the defense mechanism of plants are induced swiftly and distinctively by pathogen infection. The PCD or stress response genes include *CaLRR51* (leucine-rich repeat protein) [18], *CaDEF1* (defensin) [19], PFLP (plant ferredoxin-like protein) [20], *CaSAR8*.2 [21], *CaLOX1* (lipoxygenase) [22], *CaMBL1* (mannose-binding lectin) [23], *CaPO2* (peroxidase) [24], *CaPR1* (pathogenesis-related protein 1) [25], *CaPR10* [26], *CaChiIV1,* and *CaChiVI2* (chitinase) [27,28].

Several plant chitinases are considered to be pathogenesis-related (PR) proteins [29]. Basically, chitinase (EC 3.2.1.14) is an endo-splitting enzyme that hydrolyzes the chitin polymer (a β-1,4-linked N-acetylglucosamine), a fundamental structural element present in the cell walls of nematode eggs, a range of crustaceans, insects, and particularly fungi [30,31]. The genes responsible for plant chitinases are categorized into seven diverse classes based on the available domain(s) [16], and chitin-binding protein family members (CBP) contain one or more repeated chitin-binding domain(s) (CBD) [32]. The chitinase CBD is a small protein domain composed of almost 45 amino acid residues [33], which serves as a beneficial tag for the immobilization of protein on chitin [34,35]. The function of native CBD is to anchor chitinase to chitin during degradation [36]. After the degradation of chitin-binding domain bound chitin molecule, CBD is released and rebinds to other molecules of chitin. During the degradation of chitin by the chitinase enzyme, the CBD repeats the cycle of binding, releasing, and rebinding. The expression of chitinase genes in plant tissues is highly induced by the infection of oomycetes and fungi and the presence of chitin oligosaccharides [28,37,38], while it also responds to infection by bacteria and viruses that have no chitin or associated structures in their cell walls [39]. A recent study identified that the putative genes encoding chitin synthase enzymes are present the genome of oomycetes, such as *Phytophthora infestans* and *P. sojae* [40]. Moreover, multiple reports indicated that chitinases demonstrate antifungal activity [41] and showed that the deletion of the CBD results in the loss of hydrolytic activity or results in a loss of the antifungal activity of the chitinases [42]. However, there are very few reports that indicate that chitinases exhibit explicit antifungal activity and have a role in the hypersensitive response. The interaction between the structure and antifungal activity of chitinases is still not clearly understood.

*Colletotrichum* species are ubiquitous fungal pathogens, which cause destructive diseases in various horticultural crops throughout the world [43]. *Colletotrichum* infections lead to serious damage in several botanical structures of the host [44]. To date, at least 190 species of *Colletotrichum* have been identified, subdivided into 11 species complexes and 23 singleton species, based on molecular marker fingerprinting [45,46]. Moreover, the genomes of several *Colletotrichum* species have been sequenced [47]. Several *Colletotrichum* species are the causal agents of pepper anthracnose disease, including *C. capsici*, *C. gloeosporioides*, *C. coccodes*, and *C. acutatum* [48]. Among these, the most harmful and extensively distributed pathogen is *C. acutatum* compared with the other species [49]. This pathogen mostly attacks the green, as well as the red fruits, and can lead to lesions on pepper stems, leaves, and fruits. However, sunk necrotic tissues with concentric rings of acervuli are the typical symptoms of anthracnose infections [50]. Compared with other horticultural crops, anthracnosis in pepper has been more severe in recent years, particularly that caused by *C. acutatum* [51]. Recent studies have shown that *C. acutatum* penetrates the cuticle layers of the fruits of *Capsicum* species by developing branched and well-differentiated hyphae [52]. Once inside the cells, the fungus instantly colonizes and multiplies by producing various types of conidia from acervuli. The accumulation of hydrolytic enzymes and cell-wall degrading enzymes (CWDEs) has been hypothesized to play an essential role in the pathogenesis of *Colletotrichum* species [53,54]. These enzymes enable the pathogens to cause the deterioration of cell wall of the host plant, thus, causing tissue maceration for nutrient acquisition and colonization [55]. During plant-microbe interactions, certain plants can synthesize antimicrobial compounds to prevent pathogen infections [56]. By tackling these chemical obstacles, pathogens also seem to have evolved complex mechanisms for detoxifying or avoiding the toxic effect of defense compounds. This fungal pathogen is largely controlled by the application of synthetic pesticides, management of the plant and its environment, and genetic resistance of the host plant. Therefore, applications of fungicides and the use of integrated pest management have negative effects on health and could lead to the development of resistant strains of pathogens; they also create imbalances in the microbial community, which can be adverse for the behavior of beneficial organisms [57]. Very little information about the identity of the pathogen that causes pepper anthracnose is available. This is unfortunate, since developing resistant cultivars is the most economical and environmentally friendly method.

We identified 16 chitinase genes in pepper (*Capsicum annuum* L.) in our previous research, and a nomenclature was assigned on the basis of origin of the class and chromosomal localization [16]. All CaChi genes are induced by virulent and avirulent strains of *Phytophthora capsici* and may also participate in methyl jasmonate (MeJA) and salicylic acid (SA) regulatory mechanisms [16]. In this study, the characterization of pepper pathogenesis-related chitinase gene *CaChiIII7* is designed to examine their role in the resistance to anthracnose disease caused by *C. acutatum*. The transcription of *CaChiIII7* gene is induced to remarkable levels by infection with *C. acutatum*, while *CaChiIII7* knockdown pepper plants fail to accumulate H_2_O_2_ or undergo a hypersensitive response cell death, leading to an increase in the expansion of *C. acutatum*. The relative expression level of several defense responsive genes decreased in *CaChiIII7*-silenced plants, while the transient overexpression of *CaChiIII7*-triggered HR in pepper plant leaves is accompanied by a substantial rise in the accumulation of H_2_O_2_, stronger cell death, and also certain defense genes. Similarly, for the initiation of HR cell death, the chitinase gene *CaChiIII7* must be localized in the plasma membrane and cytoplasm of a cell. Collectively, these findings suggest that the pepper chitinase gene *CaChiIII7* is required for cell death and is a positive regulator of defense responses against microbial pathogens.

## 2. Results

### 2.1. Sequence Analysis and Characteristics of Pepper Chitinase Gene

A cDNA fragment (Capana07g001181) with the annotation of chitin-binding protein (CBP) was cloned using RNA extracted from pepper leaves of the AA3 cultivar. As reported in our previous study, the genomic sequence of *CaChiIII7* gene consists of 609 nucleotides that lack an intron, whereas the full-length CDS is 609 bp that encodes 202 amino acids (Table S1). The 1.5 kb upstream region of *CaChiIII7* from the start codon (ATG) was examined by PlantCARE [58], an online tool to predict the possibility of *cis*-acting elements that are involved in the fungal elicitor of a pathogenesis-related (PR) gene. The predicted analysis revealed that the promoter of *CaChiIII* contained a Box-W1 (TTGACC) fungal elicitor responsive element [16].

**Figure 1:**
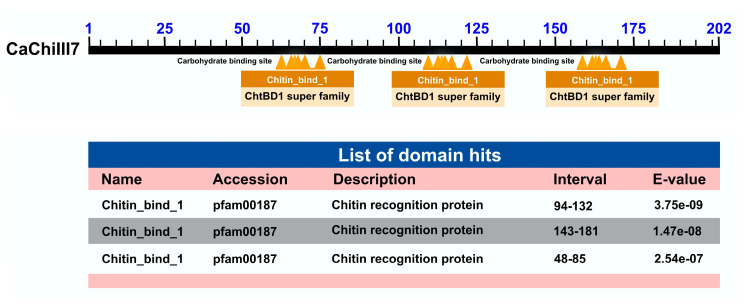
Schematic representation of the conserved domains present in the CaChiIII7 protein. The scale bar indicates the length of protein (aa). The details of conserved domains were retrieved from online tool Conserved Domain Database (CDD) [59].

Furthermore, three identical conserved domains designated chitin_binding_1 (pfam00187) have been identified in the CaChiIII7 protein; therefore, domains are located at 48–85 aa, 94–132 aa, and 143–181 aa (Figure 1). The homologs that share the same architecture and domains of the target gene in other crops are shown in Table 1. They revealed that the chitin_binding_1 domain mostly functions as an antifungal protein. The predicted gene ontology (GO) enrichment analysis of *CaChiIII7* comprised two categories, including biological process and molecular analysis (Table 2). The expected results revealed highly regulated functions that include cell wall macromolecule and polysaccharide catabolic processes, chitin degradation process, defense responses to fungi, the death of cells of other organisms, cadmium ion, and the HR. Furthermore, the predicted molecular functions of CaChiIII7 proteins suggest that they predominantly participated in chitinase, hydrolase, and glycosidase activities and served as antimicrobial and fungicidal agents.

### 2.2. Domain Assignment and Protein-Protein Interaction of an Arabidopsis Chitinase Gene

**Figure 2:**
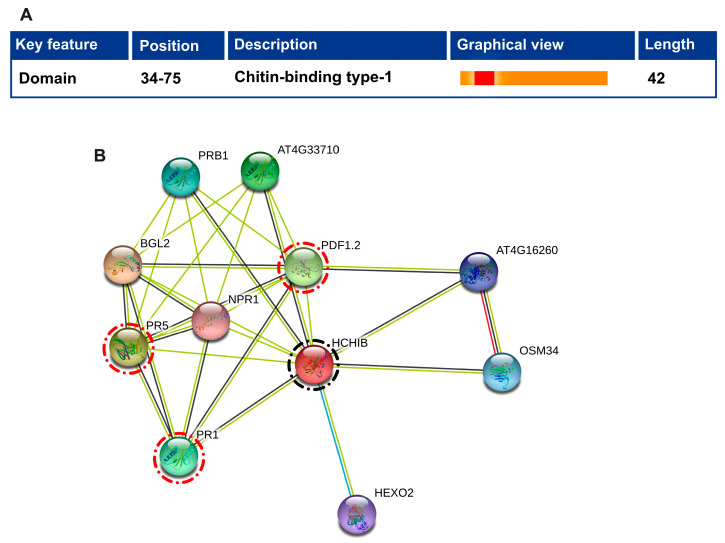
The predicted chitin-binding type 1 domain and protein-protein interaction of Arabidopsis chitinase gene ATHCHIB (homolog of pepper chitinase *CaChiIII7*). (**A**) predicted chitin-binding type 1 domain was identified using online tool Conserved Domain Database (CDD) https://www.ncbi.nlm.nih.gov/cdd [59]. (**B**) As a query sequence ATHCHIB (AT3G12500.1) was used for protein-protein interaction using the online tool STRING (https://string-db.org/). Note: the black encircled protein shows the query sequence, and the red encircled one shows other interactive defense-related proteins.

Arabidopsis is a popular model plant. Since the roles of numerous chitinase genes have been thoroughly studied in Arabidopsis, we used the chitin-binding protein gene ortholog between the pepper and Arabidopsis genomes to study the putative function of the pepper chitinase gene (*CaChiIII7*). Based on these findings, we were able to assume that the interaction with PR genes and function of *CaChiIII7* gene based on their Arabidopsis homologs, facilitating research into the roles of chitinase gene in pepper. To understand the possible role of the Arabidopsis chitinase gene (AT3G12500.1), the available domain and protein-protein interaction map were drawn using NCBI and the STRING tool (https://string-db.org/), respectively. Our analysis suggested that the Arabidopsis chitinase gene (AT3G12500.1) had one chitin-binding type 1 domain at position 34–75 (Figure 2A). In addition, the STRING analysis revealed that AT3G12500.1 has closely interacted with PR proteins (PR1, PR5, NPR1, and PRB1), defensin-like protein (PRF1.2), and beta-hexosaminidase 2 (HEXO2) proteins (Figure 2B). All these proteins played a major role in the defense mechanism of the plant, particularly when the plant interacts with pathogens. The target gene (*CaChiIII7*) may play an important role in the biotic stress response of pepper plants.

### 2.3. Subcellular Localization of the CaChiIII7 Protein

**Figure 3:**
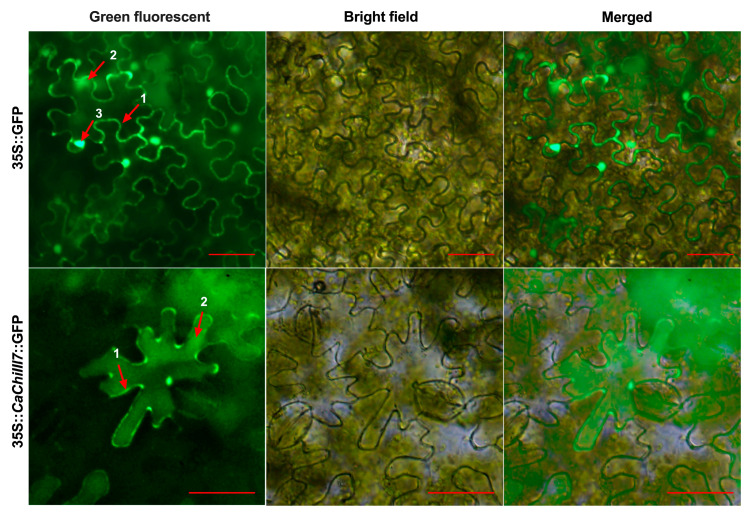
Protein localization assay of CaChiIII7. Transcript of CaChiIII7 fused with green fluorescence protein (GFP) via *Agrobacterium*-mediated transient expression in *Nicotiana benthamiana* epidermal cells. The GFP signals were visualized using a fluorescent confocal microscope (Olympus BX63) with an emission 509 nm and 488 nm excitation wavelengths (Olympus, Tokyo, Japan) after 48 h of agroinfiltration. The numbers 1, 2, and 3 represent the cell membrane, cytoplasm, and nucleus, respectively, and the red line at the bottom right corner of each picture equals 50 μm.

The ORF fragment of CaChiIII7 was recombined with the pVBG2307 (expression vector) that is composed of green fluorescence protein (GFP), a reporter gene, and a 35S promoter to establish the subcellular localization of the CaChiIII7 protein. *Agrobacterium tumefaciens* strain GV3101 with pVBG2307::GFP (used as a control) and pVBG2307::CaChiIII7::GFP vectors were rapidly expressed in the epidermal tissue of *Nicotiana benthamiana* plants. Confocal laser micrographs suggested that pVBG2307::GFP (mock) displayed GFP signals in three main components of the cell, containing the nucleus, cytoplasm, and cell membrane, while pVBG2307::CaChiIII7::GFP revealed GFP signals in the cytoplasm and cell membrane (Figure 3). This result indicated that the CaChiIII7protein is located in the cytoplasm, as well as the cell membrane of the epidermal cell.

### 2.4. Knockdown of the Chitinase Gene CaChiIII7 Attenuates the Resistance of Pepper to Colletotrichum Acutatum

**Figure 4:**
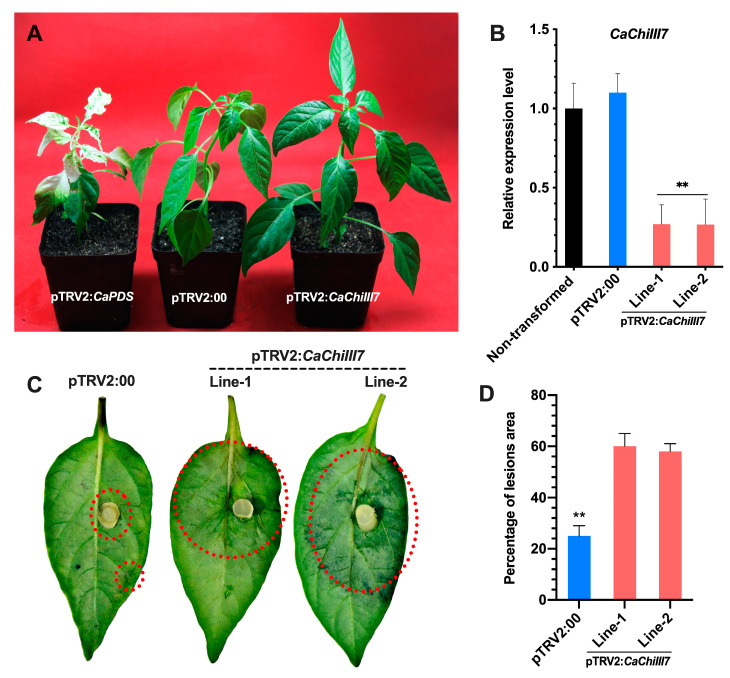
A representative phenotype and silencing efficiency analysis of *CaChiIII7* in pepper plants. (**A**) The phonotypes of pTRV2:*CaPDS* (positive control), pTRV2:00 (negative control) and pTRV2:*CaChiIII7* (*CaChiIII7*-silenced). (**B**) Silencing efficiency test of *CaChiIII7*gene in *CaChiIII7* knockdown and control (non-transformed, pTRV2:00) pepper plants. (**C**) *C. acutatum* lesions on pTRV2:00 (negative control) and pTRV2:*CaChiIII7* (*CaChiIII7*-silenced) pepper leaves. Photographs were taken 72 h after inoculation with *C. acutatum.* (**D**) The infected areas were measured using the ImageJ tool [60], and their percentages were calculated. Values are the means with standard deviations (± SD), and ** denotes highly significant differences as analyzed by a Duncan’s multiple range (DMR) test (*p* < 0.05).

The efficiency of virus-induced gene silencing (VIGS) of the *CaChiIII7*gene was verified after inoculation with *C. acutatum* in the *CaChiIII7*-knockdown (pTRV2:*CaChiIII7*) pepper plants through quantitative real-time polymerase chain reaction (qRT-PCR) analysis (Figure 4A,B). The silencing efficiency results (L_1_ = 73% and L_2_ = 74%) showed that *CaChiIII7* transcription was null or very weak in pTRV2:*CaChiIII7* (*CaChiIII7*-silenced) pepper plant leaves, indicating that the knockdown of *CaChiIII7* is highly efficient in pepper plants. However, the knockdown of this chitinase gene *CaChiIII7* in pepper plants resulted in a highly vulnerable response to infection with *C. acutatum*. Additionally, after 3–4 days of *C. acutatum* inoculation, the average diseased area of *CaChiIII7*-silenced pepper leaves of both lines (L_1_ and L_2_) showed a substantial increase in the severity of disease symptoms relative to TRV2:00 (empty vector control) leaves (Figure 4C). However, pathogen inoculation did not induce severe disease symptoms in the leaves of negative control (TRV:00) leaves, whereas TRV2:*CaChiIII7* plants exhibited noticeable necrotic symptoms. Fungal growth (hygrophanous lesions) in both lines of the *CaChiIII7* knockdown leaves was almost > 2.5-fold greater than that in TRV:00 (Figure 4D). These results implied an increase in susceptibility of pepper plants to *C. acutatum* infection owing to the loss of function of *CaChiIII7* by VIGS.

### 2.5. CaChiIII7 Interaction with Defense-Related Genes

**Figure 5:**
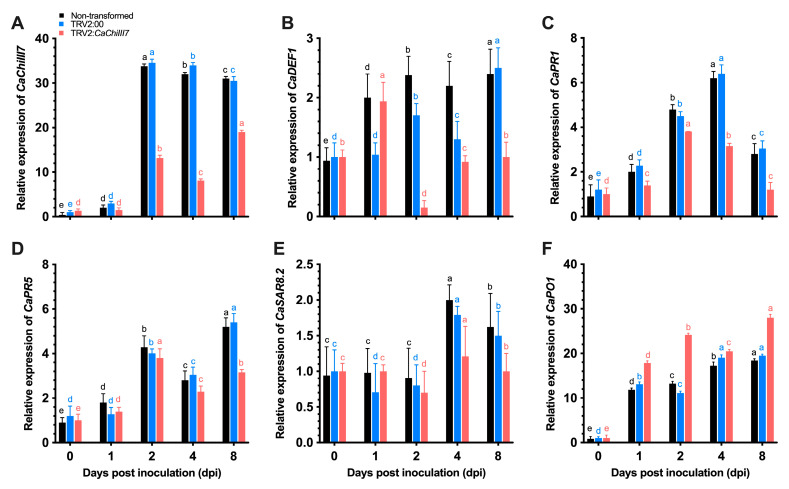
Qualitative real-time polymerase chain reaction (qRT-PCR) analyses of *CaChiIII7* transcription and defense-response genes in *CaChiIII7*-silenced (TRV2:*CaChiIII7*) and control (non-transformed, TRV2:00) pepper leaves inoculated with *Colletotrichum acutatum*. (**A**) *CaChiIII7* (chitinase); (**B**) *CaDEF1* (defensin); (**C**) *CaPR1* (pathogenesis-related 1); (**D**) *CaPR5* (pathogenesis-related; 5) (**E**) *CaSAR8.2* (systemic acquired resistance); (**F**) *CaPO1* (peroxidase). Defense genes were selected based on protein-protein interactions, Figure 2B. The control sample values were set to 1 for normalizing the transcription levels of other genes. Data are the means with standard deviations (± SD) and lower-case letters (a–e) indicate significant differences as analyzed by a Duncan’s multiple range (DMR) test (*p* < 0.05).

Following inoculation with *C. acutatum*, the transcript of chitinase gene (*CaChiIII7*) was examined in non-transformed, pTRV2:00, and *CaChiIII7*-silenced (pTRV2:*CaChiIII7*) pepper plants. A significant difference was noted in the control (non-transformed and pTRV2:00) and pTRV2:*CaChiIII7* samples at each time point, which indicated that the transcript level of *CaChiIII7* is lower in pTRV2:*CaChiIII7* relative to non-transformed and pTRV2:00 plants. However, the highest difference of 76% at 4-days post-inoculation (dpi) and > 61% at 2 dpi was observed in pTRV2:00 and *CaChiIII7*-silenced plants with values of 33.97 and 8.10 at 4 dpi, while they were 34.55 and 13.19 at 2 dpi, respectively (Figure 5A).

Furthermore, the quantitative real-time PCR assays were used to determine the interactive role of *CaChiIII7* with the expression of other defense response genes during infection with *C. acutatum* in pepper plants. The silencing of chitinase gene *CaChiIII7* did not affect the transcript of *CaSAR8.2* during the early infection period [21]. However, the transcripts of *CaDEF1*, *CaPR1*, *CaPR5,* and *CaPO1*, which are typically stimulated by *C. acutatum* infection, were reduced remarkably by *CaChiIII7* knockdown (Figure 5). The level of expression of the defensin gene (*CaDEF1*) [19] by *C. acutatum* infection during 8 dpi was elevated (2.5) in pTRV2:00 compared with pTRV2:*CaChiIII7* (1.0) (Figure 5B). Whereas the pathogenesis-related genes *CaPR1* and *CaPR5* [61] responded positively with high induction. Their level of expression in the pTRV2:00 plants was higher than that of the pTRV2:*CaChiIII7* plants at all time points examined (Figure 5C, D), which exhibited a close interaction with chitinase gene *CaChiIII7*. The transcription of *CaPO1* (peroxidase) [24] in *CaChiIII7*-silenced pepper leaves was substantially stronger than that of TRV2:00 (empty-vector control) plants at most of the time points after the *C. acutatum* inoculation (Figure 5F). Altogether, these results show that the knockdown of *CaChiIII7* fine-tunes the transcriptional regulation of defense response genes during *C. acutatum* infections.

### 2.6. Oxidative Burst and Cell Death are Compromised in CaChiIII7-Silenced Pepper

**Figure 6:**
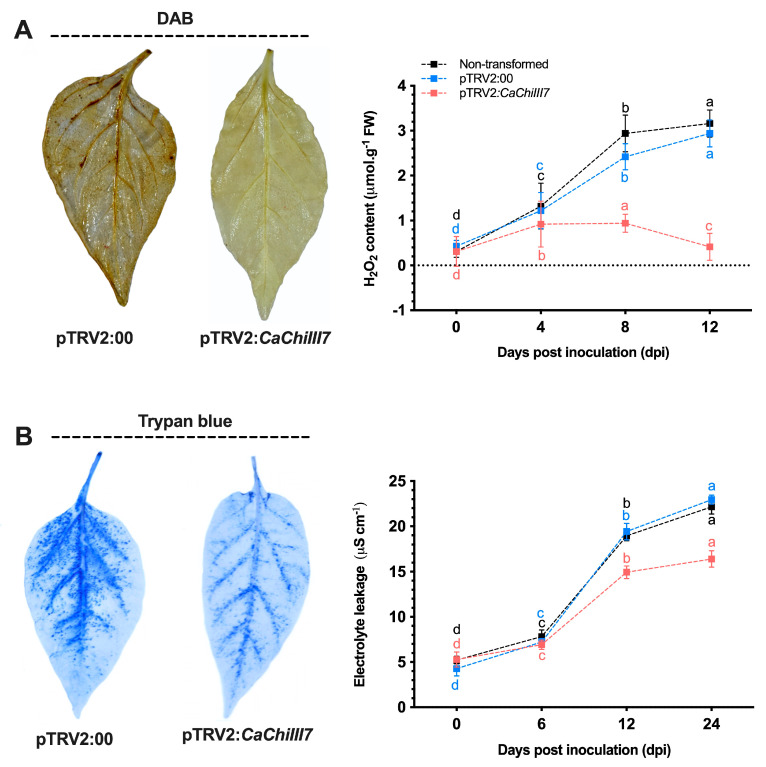
Knockdown of chitinase gene *CaChiIII7* reduces the hypersensitive response of pepper plants infected with *Colletotrichum acutatum*. (**A**) Representative photos show 3,3′-diaminobenzidine (DAB) staining and the plotted results are quantification of H_2_O_2_ in *CaChiIII7-*silenced (pTRV2:*CaChiIII7*) and empty-vector control (pTRV2:00) pepper leaves infected with *C. acutatum*. (**B**) Photos of trypan blue staining and electrolyte leakage quantification in *CaChiIII7-*silenced (pTRV2:*CaChiIII7*) and empty-vector control (pTRV2:00) leaves infected with *C. acutatum*. Data are the means with standard deviations (± SD), and lower-case letters (a–d) indicate significant differences as analyzed by a Duncan’s multiple range (DMR) test (*p* < 0.05).

The growth of *C. acutatum* significantly increased in pTRV2:*CaChiIII7* (*CaChiIII7*-silenced) pepper plants relative to that of plants expressing pTRV2:00 (empty vector) and non-transformed. However, at 1 dpi of *C. acutatum*, the production of H_2_O_2_ and cell death were clearly visualized by 3,3′-diaminobenzidine (DAB) and trypan blue staining in non-transformed, pTRV2:00 (control) and pTRV2:*CaChiIII7* pepper plant leaves. A substantial reduction in H_2_O_2_ and cell death was detected in pTRV2:*CaChiIII7* that had been inoculated with *C. acutatum* (Figure 6). The H_2_O_2_ burst during the *C. acutatum* inoculation was suppressed by *CaChiIII7* knockdown, as identified by H_2_O_2_ quantification and DAB staining (Figure 6A). However, during the early infection period, H_2_O_2_ accumulation remained close to the basal level in *CaChiIII7*-silenced plants. All of these findings validate the concept that the chitinase gene *CaChiIII7* has a vital role in the H_2_O_2_ burst and early defense signaling during *C. acutatum* infection, which plays a fundamental role in plant defense mechanisms against the pathogen.

The silencing of CaChillI7 resulted in a significant reduction of cell death at 2 dpi as quantified by trypan blue staining and an electrolyte leakage assay of the leaf discs (Figure 6B). The ion leakage in *CaChiIII7*-silenced leaves was significantly lower compared with those of the empty vector control leaves during infection of *C. acutatum*, while the highest difference (> 28%) was observed at 24 h post-inoculation (hpi). Collectively, these findings demonstrate that the chitinase response gene *CaChiIII7* plays an important function in the HR and basal defense associated with the resistance of pepper plants to *C. acutatum* infection.

### 2.7. Proline Content and Root Activity

**Figure 7:**
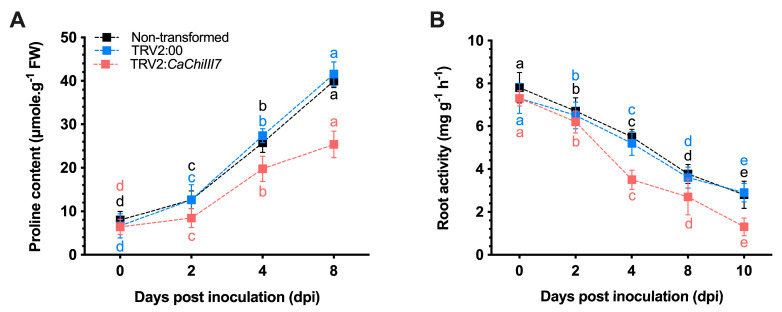
Biochemical indices of CaChiIII7-silenced and control (non-transformed and pTRV2:00) plants after *Colletotrichum acutatum* infection. (**A**) Accumulation of proline content was measured using the acid-ninhydrin method, and the absorbance of the organic phase remained steady at 520 nm. (**B**) Root activity was measured using the triphenyl-tetrazolium chloride (TTC) method. A total of 0.2 g of fresh root tips of pepper plants after inoculation with *C. acutatum* were obtained at various time points. Values are the means with standard deviations (±SD), and lowercase letters (a–e) indicate significant differences as analyzed by a Duncan’s multiple range (DMR) test (*p* < 0.05).

Proline content and antioxidant enzymes diminish the risk of oxidative damage trigger by various stresses [27,62]. The content of proline as a regulator of *C. acutatum* in the non-transformed, pTRV2:00, and *CaChiIII7*-silenced pepper plants was also measured. During stressful conditions, the concentration of proline in *CaChiIII7*-silenced plants was significantly lower than those in non-transformed and pTRV2:00 (control) plants at each time point (Figure 7A). During infection with *C. acutatum*, the accumulation of proline steadily increased, while a remarkable change was detected between *CaChiIII7*-silenced plants and the control pepper plants. However, pTRV2:*CaChiIII7* plants had a lower accumulation of proline relative to the control plants, i.e., 19.7 μmol g^−1^ fresh weight (4 dpi) and 25.4 μmol·g^−1^ FW (8 dpi), which is > 27% and > 38% lower than that of pTRV2:00, respectively. These findings reflect the relationship between the chitinase gene *CaChiIII7* and the biosynthesis of proline. The knockdown of *CaChiIII7* modulated changes in plant physiology, which might lead to discoveries of a vital role in the defense mechanism of pepper plants against *C. acutatum*.

Moreover, the vigor of the metabolism in the root system was determined by measuring root activity using the triphenyl tetrazolium chloride (TTC) method [63]. TCC was reduced in the control (non-transformed and pTRV2:00) and *CaChiIII7*-silenced plants that were treated with *C. acutatum* (Figure 7B). The root activity was reduced with the passage of time in *CaChiIII7*-silenced and control plants. However, pTRV2:*CaChiIII7* plants that were inoculated with *C. acutatum* had substantially lower root activity compared with the non-transformed and TRV2:00 control plants. The highest gap was recorded at 4 dpi, while the lowest activity was recorded in pTRV2:*CaChiIII7* plants at 10 dpi, which had 55% lower activity compared with the control plants.

### 2.8. Transient Expression of CaChiIII7 in Pepper Leaves

**Figure 8:**
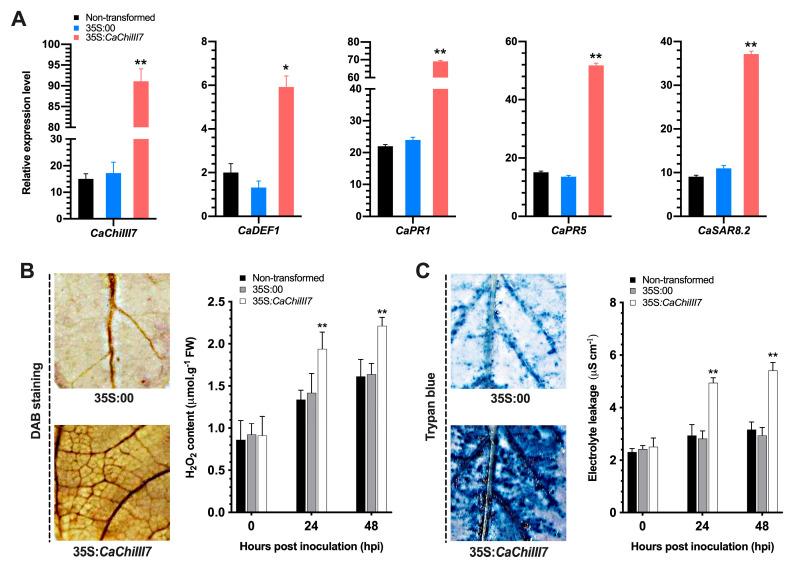
*Agrobacterium*-mediated chitinase gene *CaChiIII7* transient overexpression triggers cell death response and the activation of defense genes in pepper leaves. (**A**) The level of expression of *CaChiIII7* and other defense-related genes (*CaDEF1*, *CaPR1*, *CaPR5*, and *CaSAR8.2*) in *CaChiIII7* transiently overexpressed and control pepper plant leaves after 24 h of agroinfiltration. (Note: defense genes were selected based on protein-protein interactions, Figure 2B); (**B**) 3,3-Diaminobenzidine (DAB) staining and quantification of the accumulation of H_2_O_2_ in pepper leaves after agroinfiltration. (**C**) Trypan blue staining and the measurement of cell death in agroinfiltrated pepper leaves (0.5 cm diameter leaf discs). The electrolyte leakage caused by the cell death was examined using quantifying ion conductivity at indicated periods after agroinfiltration at the titer (OD_600_ = 1.0). Values are the means ± standard deviations (SD), and asterisk(s) denotes significant and differences as analyzed by a Duncan’s multiple range (DMR) test (*p* < 0.05).

To elucidate the function of the chitinase responsible gene *CaChiIII7*, an *Agrobacterium*-mediated transient overexpression of *CaChiIII7* in pepper leaves (in planta) regulated by the 35S promoter (cauliflower mosaic virus, CaMV) was implemented. The pepper leaves that had ectopically expressed *CaChiIII7* displayed intensive necrotic cell death symptoms after 48 h of agroinfiltration. In contrast, the transcript level of chitinase gene *CaChiIII7* increased up to > 5-fold in the leaves of 35S:*CaChiIII7* inoculated with *C. acutatum* compared with that of 35S:00 (control), whereas the transcriptional regulation of other co-expressed defensive genes, including *CaDEF1*, *CaPR1*, *CaPR5,* and *CaSAR8*.2, were also examined post-agroinfiltration (Figure 8A). As a result, the transiently expressed *CaChiIII7* gene in pepper leaves distinctly induced all the defense response genes examined. In particular, the SA-mediated marker genes, including *CaPR1* (2.9-fold), *CaPR5* (3.7-fold), and *CaSAR8.2* (3.5-fold), were strongly upregulated relative to their particular control, whereas the induction of *CaDEF1* was comparatively strong in leaves that expressed *CaChiIII7* relative to the empty vector but substantially lower than those of the other defense-related genes. The transcript levels of *CaPR1*, *CaPR5*, and *CaSAR8*.2 in leaves that expressed *CaChiIII7* increased noticeably compared with those in the non-transformed and 35S:00 (empty vector).

Furthermore, the accumulations of H_2_O_2_ and cell death were determined. The transient overexpression of *CaChiIII7* enhanced the biosynthesis of H_2_O_2_ and induced cell death. Pepper leaves that were *CaChiIII7* were stained with DAB for H_2_O_2_ biosynthesis and showed that the death of cells was preceded by an oxidative burst. The 35S:*CaChiIII7* leaves exhibited an increase in H_2_O_2_ production at 24 and 48 hpi, which is 1.94 and 2.21 μmol g^−1^ FW, respectively, whereas H_2_O_2_ was barely detected in 35S:00 (control) (Figure 8B). The cellular electrolyte leakage was determined on the basis of change in conductivity using leaf discs infiltrated via *Agrobacterium* that harbored 35S:*CaChiIII7* and 35S:00 (empty vector) (Figure 8C). Pepper leaf tissues that transiently expressed *CaChiIII7* significantly induced electrolyte leakage by increasing ion conductivity at 24 and 48 h after agroinfiltration (4.94 and 5.41 μS cm^−1^, respectively), demonstrating that the transcription of *CaChiIII7* conferred cell death in pepper leaves. Therefore, trypan blue staining confirmed the cell death in leaves that transiently expressed *CaChiIII7* (Figure 8C).

## 3. Discussion

Plants continuously encounter numerous pathogens; therefore, plants address these problems by adopting defense response strategies at many levels, such as the activation of signaling networks, regulation of secretory pathways, and induction of defense-related genes [1,2]. In these pathways, signaling molecules, such as salicylic acid (SA), jasmonic acid (JA), ethylene (ET), abscisic acid (ABA), hydrogen peroxide (H_2_O_2_), and nitric oxide (NO), are identified as secondary signals [64,65,66]. The induction of these signaling patterns can alter gene expression, resulting in specific defense responses against stress. The ability of soluble sugar (sucrose and monosaccharides) to act as a primary molecule in the regulation of phytohormones was recently identified [7]. In addition, studies have also found that sugar-induced signal transduction pathways may interact with hormonal pathways by activating a complex and extensive signal network in plant cells. Such interactions regulate metabolic processes during plant growth and development, as well as during responses to biotic and abiotic stresses [67,68,69,70]. The previous study demonstrated that sugar signaling is crucial not only for the development of plant, but it may also play a vital role to provide regulatory molecules to control plant defense mechanism to attack the pathogen through the induction of pathogenesis-related (PR) or defensive genes [10,71].

In this study, we investigated the pathogen-induced pepper gene *CaChiIII7* that encodes a chitinase enzyme as a crucial protein needed to activate defense responses against microbial pathogens. This vital protein has a repeated type 1 chitin-binding domain (ChtBD1) or hevein domain (Figure 1) [16]. This domain is found in plants and fungi, which is also referred as a lectin domain that binds N-acetylglucosamine, plant endochitinases, and wound-induced proteins such as hevein in particular. The three-dimensional structure of the hevein domain (low molecular weight) is an integral part of the IgE-binding allergen isolated from natural rubber latex and also the alpha subunit of *Kluyveromyces lactis* killer toxin. The chitin-binding domain or hevein primarily recognizes the chitin subunits that are located in the N-terminal regions to glycosyl hydrolase domains in chitinases. [59]. This CaChiIII7 is a putative chitinase protein that can share high sequence homology with other chitinases, while the homolog of this protein in Arabidopsis is ATHCHIB (AT3G12500.1). ATHCHIB belongs to a unique class of chitin-binding protein families that is a pathogenesis-related (PR) group. However, owing to its redundancies, the function of pepper chitinase gene *CaChiIII7* is not fully understood. Therefore, the functional study of the *CaChiIII7* gene may provide some insights to understand the roles of chitinase enzymes in plant innate immunity. We also found that the target gene is involved in the resistance to pepper anthracnose disease caused by *C. acutatum* using qRT-PCR, the VIGS assay, and transient overexpression.

Typically, pathogenesis-related proteins are secreted into the apoplast where they are thought to exhibit their defensive functions against pathogenic microbes [29]. Through transiently expressing CaChiIII7-GFP fusion protein in the epidermal tissue of *Nicotiana benthamiana* plants, we discovered the localization of CaChiIII7 in the plasma membrane and cytoplasm of a cell, also retained within the cell (Figure 3). As many of the pathogenesis-related proteins that recognize and interact directly with pathogens are located within the plasma membrane of a cell [72], we had hypothesized that the CaChiIII7 protein localized in the plasma membrane and cytoplasm might have a decisive role in defense response. However, the induction of an HR might require that *CaChiIII7* be active in the plasma membrane of a cell. Additionally, plasma membrane-localized CaChiIII7 can act as a defense signal regulator, possibly by fine-tuning defense signals to prolong the proteolytic degradation of membrane-bound immune receptors [73]. During the interaction between plant and pathogenic microbes, the role of plasma membrane is highly critical because it acts as a barrier between the host and pathogen. A previous study suggested that several pathogen recognition receptors (PRRs) and pathogen-associated molecular pattern (PAMP) are located in the plasma membrane of a cell [73]. Many other defense-related proteins, such as NDR1 and H^+^-ATPases, are located at the plasma membrane where they perform their vital roles [74,75].

Virus-induced gene silencing (VIGS) is an effective reverse genetic technology for the rapid knockdown of host plant genes [76,77]. Thus, the VIGS system was utilized to functionally characterize the chitinase gene *CaChiIII7* during resistance to *C. acutatum*. The successful knockdown of *CaChiIII7* resulted in an increase in susceptibility against *C. acutatum* infection and the attenuation of defense mechanism, which was accompanied by hyphal growth of *C. acutatum*, a reduced ROS burst, and the induction of defense genes. The detached leaves of *CaChiIII7*-silenced (pTRV2:*CaChiIII7*) pepper plants [28] were inoculated with *C. acutatum*. This resulted in the observation of 34% more lesions (infection) in the pTRV2:*CaChiIII7* plants compared with pTRV2:00 (control), demonstrating that the silencing of chitinase gene *CaChiIII7* greatly increased the sensitivity to *C. acutatum* infection (Figure 4C and D). Parallel results in the same crop have been observed in which the silencing of *CaChiIV1* and *CaChiVI2* decreased resistance against *Phytophthora* blight [27,28]. The knockdown of *CaChiIII7* also substantially affected the induction of other defense response genes, particularly *CaPR1*, *CaPR5* [25], and *CaDEF1* [19]. The systemic acquired resistance (SAR) and pathogenesis-related (PR) genes are induced by SA signaling when plants are exposed to biotic stress [25,78]. The basal expression of *CaPR1* and *CaPR5*, which is an SA-dependent typical marker gene, drastically decreased in *CaChiIII7* knockdown pepper plants (Figure 5C, D). Therefore, the biosynthesis of SA may be reduced *in planta*. Interestingly, in our STRING analysis, we found that the homolog of pepper chitinase protein (CaChiIII7) in Arabidopsis (ATHCHIB) primarily clustered with pathogenesis-related 1 protein (AT2G14610.1), PR-5 (AT1G75040.1), and defensin-like protein 15 (AT5G44420.1) (Figure 2) [79]. Previous research had shown that the chitin-binding domain (CBD) has a cysteine and hinge region, which is saturated by glycine and proline. In our study, the *CaChiIII7*-silenced plant showed a significant decreased in proline accumulation at 4 and 8 dpi (Figure 7A). In a previous study, Schoöffl et al. (1999) [62] investigated that proline biosynthesis reduces the amount of ROS damage; however, in most crops, proline is considered as one of the most common compatible osmolytes to adjust the cellular osmotic pressure that is caused by a water deficit, high salinity, and other stresses [80,81,82]. Additionally, to further elucidate the role of *CaChiIII7* in the resistance of pepper against pathogen infection, root activity assays were performed to detect the negative effect of *C. acutatum* infection on *CaChiIII7* knockdown pepper plants. When the infection period was extended, the root activity in the *CaChiIII7* knockout plants decreased relative to that of pTRV2:00 and non-transformed plants, while the activity at 8 dpi was reduced remarkably (Figure 7B). Such results indicated that *CaChiIII7*-silencing decreased the resistance of pepper plants against anthracnose disease as compared with both controls. All these results from the VIGS study demonstrate that not only the *CaChiIII7* gene itself but also other defense-associated genes might be crucial for the protection of pepper plants against numerous pathogens.

Furthermore, *CaChiIII7* was transiently overexpressed in pepper leaves to verify whether *CaChiIII7* is involved in the accumulation of H_2_O_2_. The pepper leaves that overexpressed *CaChiIII7* accumulated more H_2_O_2_, had more cell death, and exhibited a significant up-regulation of defense-related genes, such as *CaPR1*, *CaPR5*, *CaDEF1,* and *CaSAR8.2* (Figure 8). Previous studies suggested that cellular oxidative bursts induced cell death and increased ion conductivity in plant cells [83,84]. Thus, we hypothesized that the chitinase proteins may coordinate with other defensive genes to sense the infection and participate in triggering a HR (increased ROS concentration), which prevents the extension of fungal hyphae or degrades the fungal cell wall. However, it is not clear how the gene has contributed to cell death and the reduced fungal expansion in a plant, and the relationship of *CaChiIII7* to other defense-related genes and their involvement in biotic stress resistance merits further study. Additionally, the transient overexpression of the chitinase gene *CaChiIII7* in pepper plants triggers specific defense-related genes (Figure 8A). The basal transcription of PR genes, which are typical SA-dependent marker genes [25,78], increased dramatically in pepper plants that transiently overexpressed *CaChiIII7*. Consequently, SA synthesis may be triggered *in planta*. Furthermore, the gene ontology (GO) analysis revealed that the chitinase genes, such as *CaChiIII7*, are involved in the defense mechanisms of plants, such as antimicrobial activity, the production of antifungal compounds, and HR (Table 2). These findings parallel those of our previous study that found that chitin-binding protein family members are primarily involved in the resistance of pepper plants [16]. Altogether, these findings provide evidence for the concept that *CaChiIII7* participates in the HR and immune response of pepper plants to counter anthracnose disease caused by *C. acutatum*.

**Figure 9:**
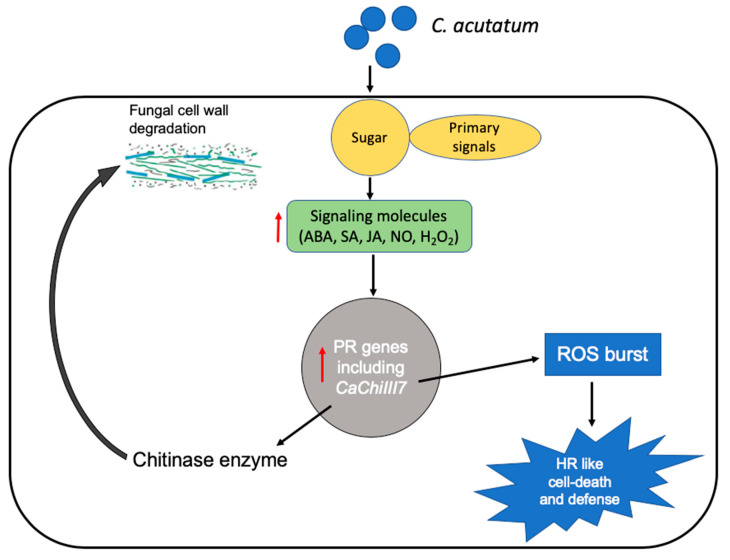
A model proposed for the chitinase gene *CaChiIII7* during innate immunity against infection by *Colletotrichum acutatum*.

Based on a previous study and current research, a novel working model is suggested for the function of the cytoplasm- and plasma membrane-localized pepper chitinase gene *CaChiIII7* to participate in the defense response and cell death (Figure 9). The identification of primary signals of the pathogen, such as sucrose and monosaccharides, that regulate the level of signaling molecules (ABA, SA, JA, ET, and H_2_O_2_) and enhance defense-related genes provides suggestions for avenues of further study [7]. Among all the defensive proteins, chitinase is an important antifungal protein generated by plants whose function is to exhibit antifungal activity by degrading the chitin that is a vital component of the fungal cell wall [85,86]. After inoculation with *C. acutatum*, the chitinase gene *CaChiIII7* is rapidly expressed to trigger defense responses by expressing defense genes, such as *CaPR1*, *CaPR5*, *CaDEF1*, *CaSAR8.2*, and *CaPO1*. In addition, early cell death can be caused by a burst of ROS (ROS accumulation), possibly with the help of the damage-associated molecular pattern (DAMP) and effector-triggered immunity (ETI) recognition receptors. Thus, *CaChiIII7* promotes ROS biosynthesis and defense-based gene transcription that can hinder the inhibitory role of *C. acutatum* effector proteins and eventually lead to the initiation of HR cell death and plant defense against the pathogen.

## 4. Materials and Methods

### 4.1. Plant Materials and Inoculum Preparation

Pepper (*Capsicum annuum* L. cultivar AA3) plants were grown in a growth chamber at ±28 °C, with a photoperiod of 16 h at a light intensity of 70 μmol photons m^−2^ s^−1^. The pure isolate of *Colletotrichum acutatum* was obtained from the same lab repository. The pure spores of *C. acutatum* were shaken in liquid potato dextrose agar (PDA) media for 72 h at ±28 °C. The filtered suspension was centrifuged at 4000 rpm for 5 min, and the spores obtained were washed three times with deionized water. The concentration (2 × 10^5^ conidia/mL) of microspores was calculated before inoculation [87].

### 4.2. Cloning of CaChiIII7 and Web-Based Analysis

The open reading frame (ORF) of chitinase gene *CaChiIII7* was amplified from the cDNA of pepper samples with primers *CaChiIII7*-F and *CaChiIII7*-R (Tables S1 and S2). A total volume of 50 μL PCR reaction was performed, which contained 5 μL of 10 × PCR buffer, 1 μL of each primer (10 μmol L^−1^), 1 μL of dNTP (10 mM, each), 0.4 μL Taq DNA polymerase enzyme (2.5 U μL^−1^), 4 μL cDNA (50 ng μL^−1^), and 37.6 μL of ddH_2_O. The following were the PCR conditions: an initial denaturation at 94 °C for 5 min, afterward 30 cycles of 94 °C for 30 s, 58 °C for 1 min and 72 °C for 40 s, then a final extension at 72 °C for 10 min and 4 °C.

The protein sequence of the cloned chitinase gene *CaChiIII7* was BLAST into the Conserved Domain Database (CDD) https://www.ncbi.nlm.nih.gov/cdd [59] for the prediction of conserved domains. The online tool SmartBLAST (https://blast.ncbi.nlm.nih.gov/smartblast/smartBlast.cgi) was used to identify the homologs. A sequence of AT3G12500.1 was retrieved using the Arabidopsis information resource (TAIR) (https://www.arabidopsis.org/index.jsp). The online tool STRING (https://string-db.org/) [79] was used for the identification of interacting protein analyses, while the UniProt (https://www.uniprot.org/) [88] tool was used for gene ontology (GO) analyses. 

### 4.3. RNA Extraction and Quantitative Real-Time PCR (qRT-PCR) Analysis

Using collected pepper leaf samples, total RNA was extracted using the TRIzol reagent (Invitrogen, Carlsbad, CA, USA) according to the manufacturer’s instructions. RNA was purified without contaminating DNA using RNase-free DNase I. The cDNA was synthesized using the Prime-Script^TM^ RT Reagent Kit (TaKaRa, Dalian, China). NanoDrop was used to adjust the quality and quantity of cDNA. To measure the level of expression of *CaChiIII7* during infection with *C. acutatum*, qRT-PCR was performed with gene-specific primers as shown in Table S2, and the qRT-PCR analysis was conducted by a 20 μL volume system using SYBR Green PCR master mix (TaKaRa). The ubiquitin-conjugating protein gene (*CaUbi3*) was used as an internal control [89] (Table S2). The relative transcript levels of the chitinase gene (*CaChiIII7*) were computed using the 2^− ΔΔCT^ method [90].

### 4.4. Subcellular Localization of the CaChiIII7 Protein

The ORF of CaChiIII7 was fused with GFP (GFP::CaChiIII7) (Table S3) and transferred into a pVBG-2307+GFP vector (Figure S1) driven by a 35S promoter (CaMV) for transient expression. Tobacco epidermal cells were used for the subcellular analysis of the fused protein. The competent GV3101 cells were harvested and dissolved in 200 μM acetosyringone, 10 mM MES (pH 5.5), and 10 mM MgCl_2_ and injected into 4-week-old leaves of *Nicotiana benthamiana* through a needleless syringe. The tobacco plants were grown in darkness for two days and then in the growth chamber for three days. Tobacco epidermal cells were inspected under a fluorescent confocal microscope (OLYMPUS BX63) with an emission 509 nm and 488 nm excitation wavelength (Olympus, Tokyo, Japan).

### 4.5. Virus-Induced Gene Silencing (VIGS) of CaChiIII7

For the VIGS assay, the *CaChiIII7* gene was silenced using the method described by Wang et al. (2013) [91]. The amplified target fragment of 255 bp (primer pairs Table S4) of *CaChiIII7* was precisely cloned into the TRV2 vector (Figure S2) using a set of restriction enzymes (*Eco*RI and *Xho*I). The vector cassette was transformed into an *A. tumefaciens* strain (GV3101) as described by Wang et al. (2013) [92]. The positive clones were grown on rifampicin, gentamicin, and kanamycin (RGK) media. A suspension culture with OD_600_ = 1.0 was injected into the fully expanded cotyledonary leaves of pepper plants through a syringe without a needle [27,93]. Moreover, the negative control TRV2:00 (empty vector) and the positive control TRV2:*CaPDS* (phytoene desaturase) were also transformed. The plants were maintained in a growth chamber, and samples were collected from *CaChiIII7*-silenced and control plants after 45 days. For statistical analyses, a Duncan’s multiple range (DMR) test was performed using SPSS 25.0 (SPSS, Inc., Armonk, NY, USA) to evaluate the data collected at P-values ≤ 0.05. In addition, the means and their standard deviations (±SD) were graphed using GraphPad Prism 8.0 (GraphPad Software, Inc., La Jolla, CA, USA).

### 4.6. Agrobacterium-Mediated Transient Expression

Pepper plant leaves were used to conduct transient overexpression by infiltration with *A. tumefaciens* strain GV3101, including the vectors expressing *CaChiIII7* (35S:*CaChiIII7*) and mock vector as a control (35S:00), as described in the previous study [94]. The leaves were suspended in 10 mM MES (200 mM acetosyringone, pH 5.7), and the bacterial suspension (OD_600_ = 0.8–1.0) was injected in the lateral veins of fresh pepper leaves through a needleless syringe. The overexpressed *CaChiIII7* transient and control pepper leaves were used for DAB and trypan blue staining after 48 h of agroinfiltration. The photographs were taken using a Nikon D5500 camera (Nikon Corporation, Bangkok, Thailand).

### 4.7. Measurement of Contributing Attributes

To quantify the physiological attributes, the pTRV2:*CaChiIII7* (*CaChiIII7*-silenced), 35S:*CaChiIII7* (transiently expressing *CaChiIII7*), and control (pTRV2:00, 35S:00 and non-transformed) pepper plant tissues were collected at different time points. The assessment of proline content was conducted as described by Bates et al. (1973) [95]. An aqueous extract was mixed with glacial acetic acid and acid ninhydrin (2 mL each) reagent (1.25 g of ninhydrin, 30 mL of glacial acetic acid, and 20 mL of 6 M orthophosphoric acid) and heated at 100 °C for 30 min. After cooling, the reaction mixture was partitioned against toluene (4 mL), and the absorbance of the organic phase remained steady at 520 nm. The resulting values were compared with a standard curve (Sigma-Aldrich, St Louis, MO, USA). To quantify the electrolyte leakage, pepper leaf discs that were 0.5 cm in diameter were washed for 30 min in double distilled sterile water, followed by incubation for 2 h at room temperature with gentle agitation [94]. Electrolyte leakage from the leaf samples was quantified by measuring ion conductivity using an ion leakage meter. H_2_O_2_ was assayed as described by Patterson et al. (1984) [96]. The data for H_2_O_2_ were recorded at 0, 4, 8, and 12 dpi of *C. acutatum* and graphed. The absorbance was monitored at 560 nm, and H_2_O_2_ levels were calculated using a standard curve derived from a standardized solution of H_2_O_2_. However, the activity in root was measured using triphenyl-tetrazolium chloride (TTC) as described by Ou et al. (2011) and Wang et al. (2013) [97,98]. Approximately 0.2 g of fresh root tips from pepper plants that had been inoculated with *C. acutatum* were obtained at various time points (0, 2, 4, 8, and 10 dpi). Root samples were washed with ddH_2_O and dried slightly with moisture absorbent paper. A slightly modified TTC method was used to quantify the root activity, while the data were calculated using three independent biological replicates. The DMR test was performed for statistical analysis to evaluate the collected data at *p*-values ≤ 0.05. The means and their standard deviations (±SD) were graphed.

### 4.8. Detached Leaf Assay and Histological Observation

A 5 mm mycelium plug of fungus (*C. acutatum*) from an actively growing plate was inoculated into the center of the detached pepper leaves of both *CaChiIII7*-silenced (pTRV2:*CaChiIII7*) and control (pTRV2:00) plants. To maintain high relative humidity, the petri dishes were promptly sealed with parafilm and incubated at 28 °C. The ImageJ tool was used to measure the infected area/hyphal extension and quantify the degree of infection [60]. The pathogen-infected leaves were evaluated and photographed at 72 hpi.

The accumulation of H_2_O_2_ was observed by placing the pathogen-infected leaves in 1 mg mL^−1^ of DAB solution for 15 h. This was followed by the removal of chlorophyll from the stained leaves by boiling the samples in 95% absolute ethanol. In addition, the cell death of the healthy leaves and those inoculated with the pathogen was monitored by trypan blue staining. The lactophenol-trypan blue solution (lactic acid and glycerol 10 mL each, 10 g phenol, and 10 mg trypan blue mixed in 10 mL of ddH_2_O) was used to stain the pepper leaves, while a chloral hydrate solution (2.5 g mL^−1^ chloral hydrate) was used to de-stain them. The photographs were taken with a Nikon D5500.

## 5. Conclusions

Briefly, our results favor the concept that *CaChiIII7* may significantly contribute to innate immunity in pepper plants. Early responses to infection by fungal pathogens include the accumulation of ROS and transcriptional activities of PR genes as a consequence of PAMP-triggered immunity [73,84]. Transient overexpression and VIGS analyses in pepper plants indicated that chitinase gene *CaChiIII7* regulates the ROS burst, as well as the level of transcripts of defense response genes. The *CaChiIII7* protein is primarily located in the cytoplasm and plasma membrane. Based on this, we hypothesized that the protein is vital during interactions between the plant and microbe, because plasma membranes function as impediments among the pathogen and host plant. *CaChiIII7* positively regulates plant defense responses and cell death. More research work is needed to clarify how *CaChiIII7* modifies and affects physiological functions. Our findings may help to establish available avenues for plant breeding strategies aimed at the resistance of fungal pathogens to improved plant immunity.

## Figures and Tables

**Table 1:** Identification of chitin-binding domain(s) and their function in different crops

Identifier	Description	Organism	Length
P152312	Root-specific lectin	*Hordeum vulgare*	212
1611467A	Root-specific lectin	*Hordeum vulgare* subsp. vulgare	212
AAA32969	Lectin precursor	*Hordeum vulgare*	212
AAB18152	Antifungal protein	*Ipomoea nil*	91
AAA98238	Antifungal protein	*Ipomoea nil*	91
P02876	Agglutinin isolectin 2	*Triticum aestivum*	213
AAA34258	Agglutinin isolectin D precursor	*Triticum aestivum*	213
P81591	Antimicrobial protein PN-AMP1	*Ipomoea nil*	41
2UVOB	Chain B, high resolution crystal structure of wheat germ agglutinin in complex with N-acetyl-D-glucosamine	*Triticum aestivum*	171

**Table 2:** The predicted gene ontology (GO) analysis of chitinase gene *CaChiIII7*.

Gene Ontology Analysis	
Biological Process	Molecular Function
Cell wall macromolecule catabolic process	Chitinase activity
Chitin catabolic/degradation process	Chitin binding
Defense response to fungus	Antimicrobial
Killing of cells of other organisms	Hydrolase
Hypersensitive response	Glycosidase
Polysaccharide catabolic process	Fungicide
Response to cadmium ion	
Carbohydrate metabolism	
Plant defense	

## Supplementary Materials

The following are available online at https://www.mdpi.com/1422-0067/21/18/6624/s1, Figure S1: Subcellular localization vector pVBG2307-GFP, Figure S2: Virus induced gene silencing (VIGS) vector, Table S1: The CDS sequence of chitin-binding protein gene CaChiIII7 with accession number, Table S2: Primer pairs for qRT-PCR, Table S3: Primer pairs for subcellular localization of CaChiIII7, Table S4: Primer pairs for knockdown.
