# Supplementary material for: Chitinase Gene Positively Regulates Hypersensitive and Defense Responses of Pepper to Colletotrichum acutatum Infection
Source: Int J Mol Sci. 2020 Sep 10;21(18):6624. doi: 10.3390/ijms21186624 (PMC7555800; doi:10.3390/ijms21186624)
Supplement: Supplementary file 1 [file ijms-21-06624-s001.zip › Supplementry files/Supplementary Tables.docx]

**Table S1. The CDS sequence of chitin-binding protein gene *CaChiIII7* with accession number**

| ***CaChiIII7* (Capana07g001181)** |
| --- |
| ATGAAAGAAACTGCATTAATTAGCGTTTTAGCTCTAGCTTTATTCCTCCTTAAGGTCTCAGCCAAAACATCTCTTCCCTTTCATTTGTCAGACAGTTCAGCATCATCATCATCAGCCAAGCTATCAGGCTTACCGACCCTATGCGGAGAAGCTGTTGGTGGTAGAGAATGCCCTGATGGGAAGTGTTGTAGTCTAGAGGGTTATTGTGGAACAGGAGAAGCCTACTGTGCTCCTGAAAACTGTCAAAGTAATTGCGATGAGCCACCAGAGCCAGAGCTAGAATGCGGAGATCAAGCTGGTGGTAAAGAATGTCCTAATGGAGAGTGTTGTAGTATATTTGGTTCGTGTGGAACCACAGAAGACCATTGTTGGAAACCTTTTTGTCAAAGTCAGTGCAATGAGCCACCATTGCCACCAGAGCCAGAGAGATGCGGAAGGGAAGGTGGTGGTAAAGAATGTCCTACGGGAGAGTGTTGTAGTGTATTTGGTTCCTGTGGAACCACAGAAGAACATTGTGAGGAACCTTTTTGTCAGAGTCAGTGCAAAACTAGTCTAAACAAGAATCGCATGCTGAGAGGCACTCGAAGCTTCTTCCTCAATGCTCTCTAA |
| **CaChiIII7 (Protein)** |
| MKETALISVLALALFLLKVSAKTSLPFHLSDSSASSSSAKLSGLPTLCGEAVGGRECPDGKCCSLEGYCGTGEAYCAPENCQSNCDEPPEPELECGDQAGGKECPNGECCSIFGSCGTTEDHCWKPFCQSQCNEPPLPPEPERCGREGGGKECPTGECCSVFGSCGTTEEHCEEPFCQSQCKTSLNKNRMLRGTRSFFLNAL |

**Table S2. Primer pairs for qRT-PCR**

| **Gene name** | **Primer sequence (5'→3')** | **Product length** |
| --- | --- | --- |
| *CaChiIII7* | F: TCAGCCAAAACATCTCTTCCC | 121 |
|  | R: CATCAGGGCATTCTCTACCAC |  |
| *CaPR1* | F: TGGAGACTGCAGGATGCAACACT | AF053343.2 |
|  | R: TACCACCCATTGTTGCACCGAAC |  |
| *CaPO1* | F: CCCTTCAATCGACGCATCCTTTC | AF442386.1 |
|  | R: CCAAGAAATCCCCTGAGCCCTA |  |
| *CaDEF1* | F: CAAGGGAGTATGTGCTAGTGAGAC | AF442388.1 |
|  | R: TGCACAGCACTATCATTGCATAC |  |
| *CaSAR8.2* | F: CAGGGAGATGAATTCTGAGGC | AF442386.1 |
|  | R: CATATGAACCTCTATGGATTTCTG |  |
| *CaUbi3* | F: TGTCCATCTGCTCTCTGTTG | AY486137.1 |
|  | R: CACCCCAAGCACAATAAGAC |  |

F: Forward primer R: Reverse primer

**Table S3. Primer pairs for subcellular localization**

| **Primer Name** | **Primer sequence (5'→3')** | **Enzymes** |
| --- | --- | --- |
| *CaChiIII7* | F: CGGGATCCAGACTGAATGTCATAACTAACAA | *BamH1* |
|  | R: GGTACCGAGAGCATTGAGGAAGAAG | *Kpn1* |

**Table S4. Primer pairs for knockdown**

| **Primer Name** | **Primer sequence (5'→3')** | **Enzymes** |
| --- | --- | --- |
| *CaChiIII7* | F: CCGGAATTCGAAGCCTACTGTGCTCCTGA | *EcoR1* |
|  | R: CCGCTCGAGCTCTCCCGTAGGACATTCTTT | *Xho1* |
| *CaPDS* | F: GGGGAATTCTGTTGTCAAAACTCCAAGGTCTGTA | *EcoRI* |
|  | R: GGGGGATCCTTTCTCCCACTTGGTTCACTCTTGT | *BamH1* |
