# Supplementary material for: Chitinase Gene Positively Regulates Hypersensitive and Defense Responses of Pepper to Colletotrichum acutatum Infection
Source: Int J Mol Sci. 2020 Sep 10;21(18):6624. doi: 10.3390/ijms21186624 (PMC7555800; doi:10.3390/ijms21186624)

**Figure S1.** Subcellular localization vector pVBG2307-GFP


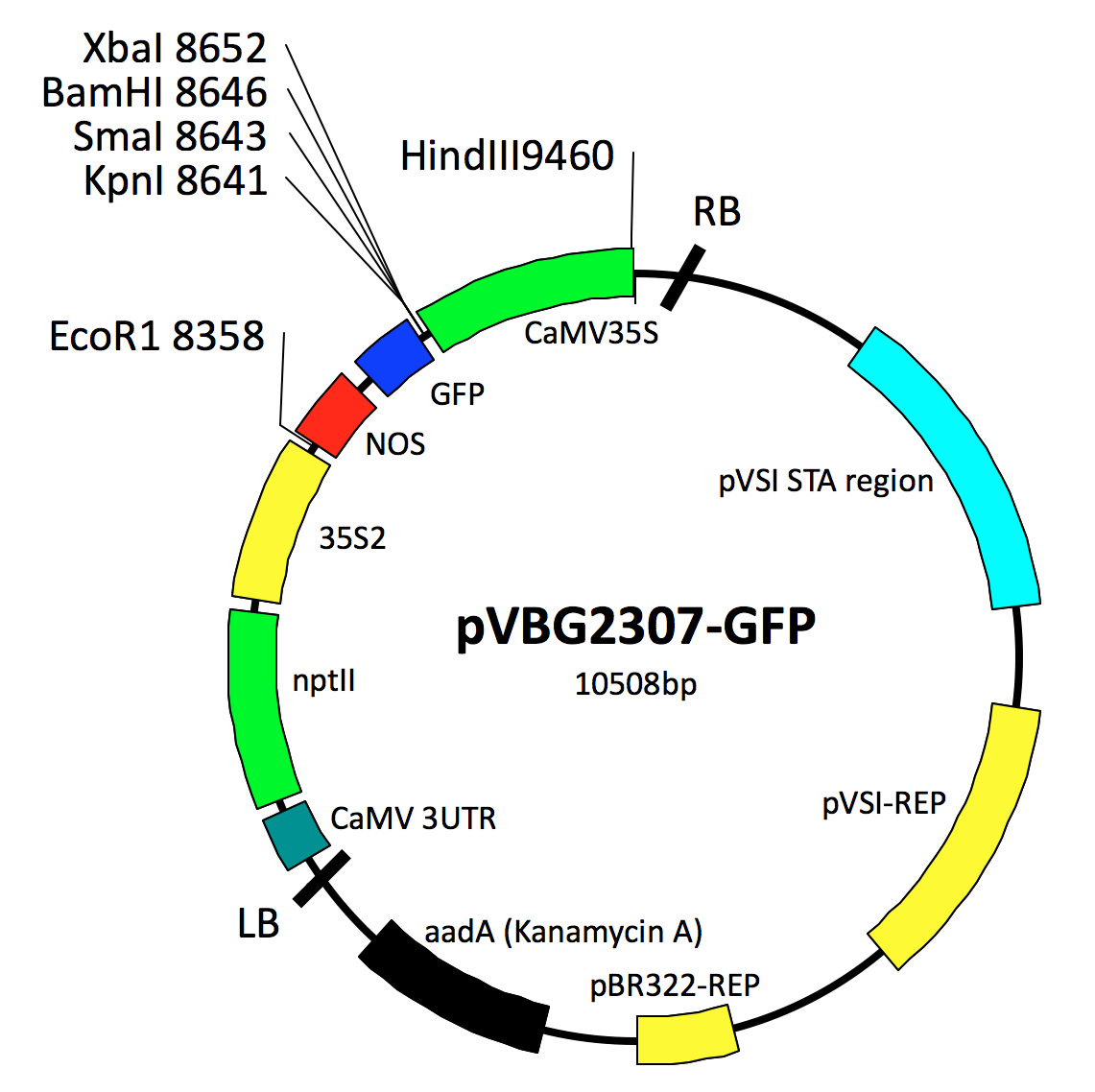


**Figure S2.** Virus induced gene silencing (VIGS) vector.


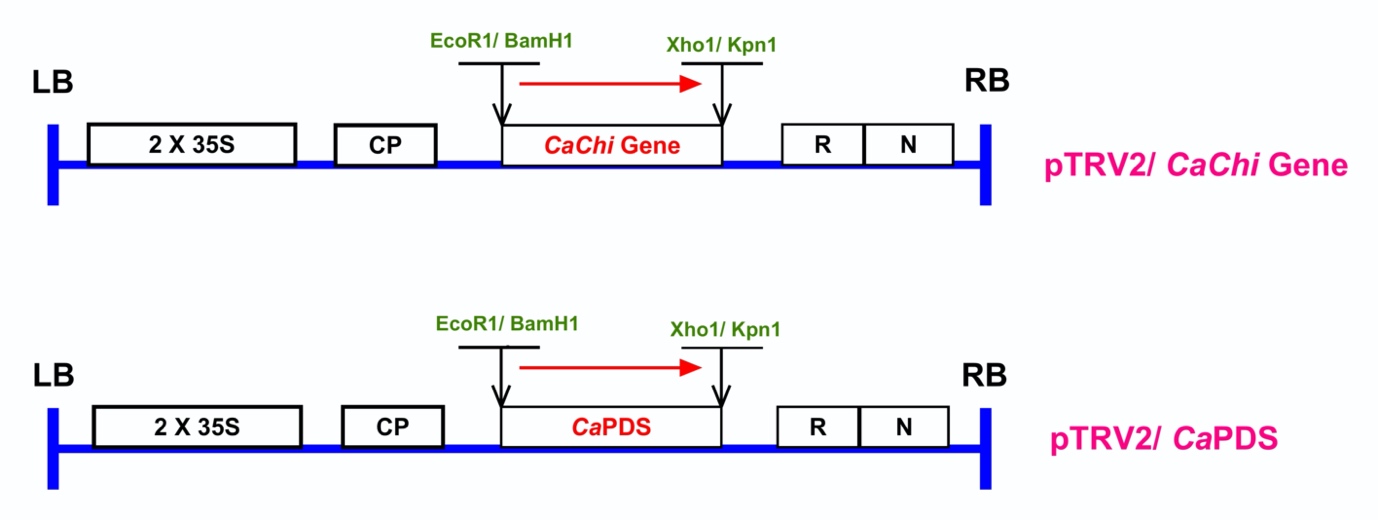

Supplement: Supplementary file 1 [file ijms-21-06624-s001.zip › Supplementry files/Suplementary Figures.docx]
